# Redundancy of protein disulfide isomerases in the catalysis of the inactivating disulfide switch in A Disintegrin and Metalloprotease 17

**DOI:** 10.1038/s41598-018-19429-4

**Published:** 2018-01-18

**Authors:** Sebastian Krossa, Axel J. Scheidig, Joachim Grötzinger, Inken Lorenzen

**Affiliations:** 1 Centre of Biochemistry and Molecular Biology, Structural Biology, Kiel University, Am Botanischen Garten 9, 24118 Kiel, Germany; 2 Institute of Biochemistry, Kiel University, Rudolf-Höber Str. 1, 24118 Kiel, Germany

## Abstract

A Disintegrin and Metalloprotease 17 (ADAM17) can cause the fast release of growth factors and inflammatory mediators from the cell surface. Its activity has to be turned on which occurs by various stimuli. The active form can be inactivated by a structural change in its ectodomain, related to the pattern of the formed disulphide bridges. The switch-off is executed by protein disulfide isomerases (PDIs) that catalyze an isomerization of two disulfide bridges and thereby cause a disulfide switch. We demonstrate that the integrity of the CGHC-motif within the active site of PDIs is indispensable. In particular, no major variation is apparent in the activities of the two catalytic domains of PDIA6. The affinities between PDIA1, PDIA3, PDIA6 and the targeted domain of ADAM17 are all in the nanomolar range and display no significant differences. The redundancy between PDIs and their disulfide switch activity in ectodomains of transmembrane proteins found *in vitro* appears to be a basic characteristic. However, different PDIs might be required *in vivo* for disulfide switches in different tissues and under different cellular and physiological situations.

## Introduction

Post-translational protein modifications are typical responses to changing environmental parameters and enable organisms to maintain their metabolic and physiological homeostasis. Ectodomain shedding is a rapid and irreversible mechanism involved in the release of growth factors and proinflammatory mediators for regeneration and immune defense. In mammalian cells, the transmembrane metalloprotease A Disintegrin and Metalloprotease 17 (ADAM17) plays a key role^1–5^ and is associated with a variety of physiological and pathophysiological processes. The latter are a consequence of the uncontrolled activity of the usually tightly controlled enzyme. A prerequisite for ADAM17 activity is its activation. This can be achieved by various signals, such as histamine, thrombin, endotoxins, or growth factors^6–8^. A crucial step in the activation is the flip of phosphatidylserine (PS) from the inner to the outer leaflet of the plasma membrane, so that the extracellular part of ADAM17 can bind to PS head groups^9^. The PS-binding motive of ADAM17 has been identified in the membrane-proximal domain (MPD), which is located C-terminal of the catalytic and disintegrin domains. Together with the directly adjacent stalk region, the so-called Conserved ADAM seventeeN Interaction Sequence (CANDIS)^10–12^, the MPD forms a functional unit that is essential for the regulation of the shedding activity by mediating substrate recognition and membrane binding^9,13–15^. Membrane binding is thought to orientate the catalytic domain in close proximity to the plasma membrane where the proteolytic cleavage of its substrates takes place^3,4,16,17^.

Two different conformations of the MPD of ADAM17 can be distinguished. The open conformation (opMPD) is a linear arrangement of two disulfide bridges (C600-C630; C635-C641), which allow PS- and substrate-binding, both of which are required for shedding activity^11^. In its closed structure (clMPD), however, the two disulfide bridges are isomerized in an overlapping order (C600-C635; C630-C641), thereby abrogating flexibility in the MPD. As a result, PS head groups and substrates are no longer accessible to the MPD-CANDIS unit, and consequently, the shedding is inactivated^9,11^. The isomerization is catalyzed by extracellularly located protein disulfide isomerases (PDIs), such as PDIA1 (PDI) and PDIA6 (Erp5)^11,18–20^. Although PDIs are endoplasmic reticulum (ER)-resident proteins having their classic function in oxidative protein folding by assisting the formation of the correct disulfide bridges^21^, they are also well established as being present at the cell surface^22–27^. Here, they reduce disulfide bridges in the ectodomains of various proteins and thus change their structure and modify their function. Consequently, PDIs are additionally involved in the regulation of cell adhesion, proteolytic accessibility, enzymatic activity, and virus entry^11,20,23,27–30^. PDIs activate integrins and thereby support coagulation and stabilize the thrombus^31–36^. In this process, active PDIs such as PDIA1, PDIA3 (Erp57), and PDIA6 are secreted from activated epithelial cells and thrombocytes^34,36–38^. In contrast to integrins, ADAM17 is inactivated by PDIs. To date, PDIA1 and PDIA6 are described as inactivating ADAM17 by the disulfide switch^11,20^. Here, we have used recombinant purified proteins to analyze the principal requirements of PDIs when catalyzing the disulfide switch.

## Results and Discussion

### Redundancy of PDIs in the catalysis of the disulfide switch

**Figure 1:**
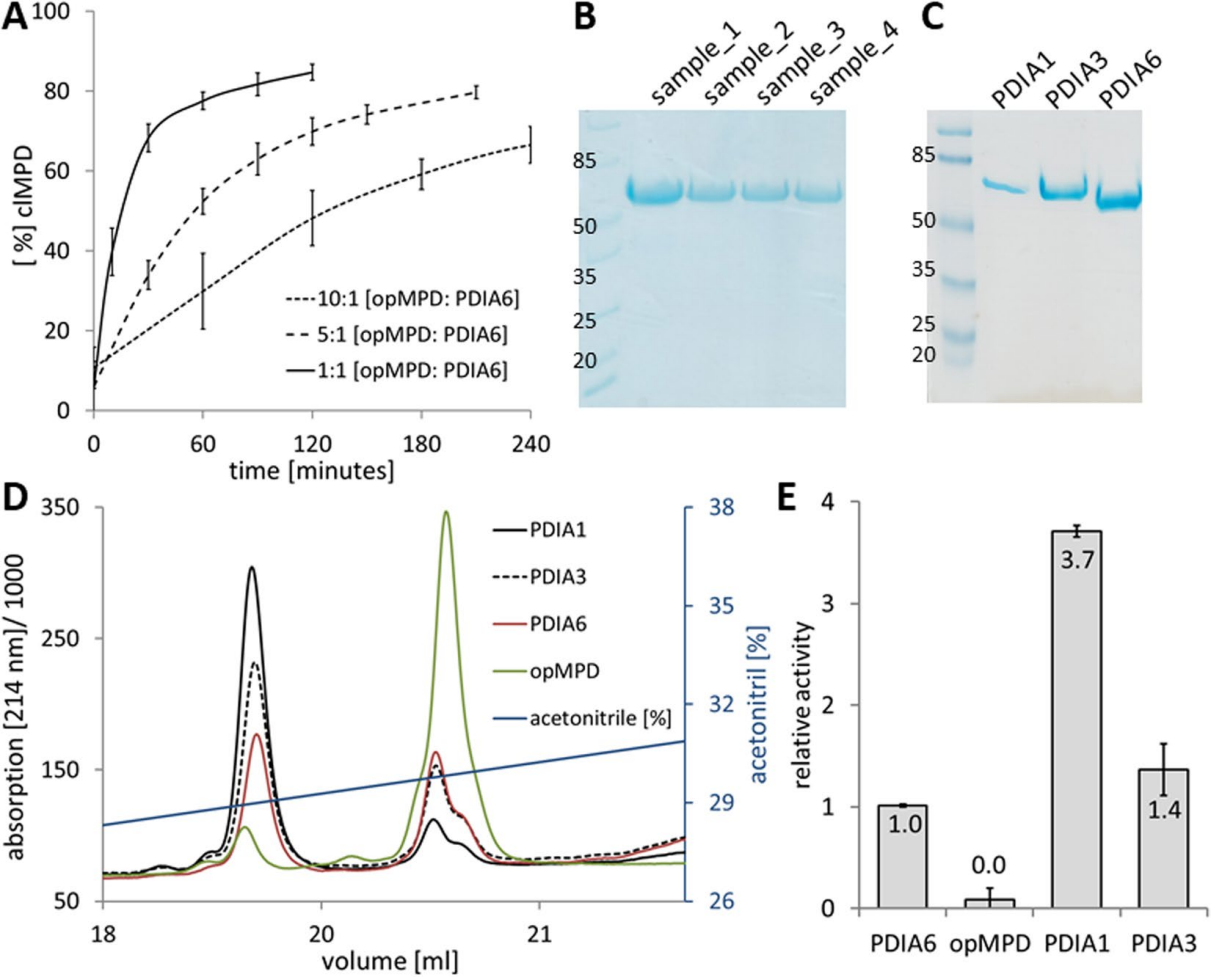
Catalytic activity of recombinant PDIs when performing isomerization of opMPD to clMPD. (**A**) Three time curves of the disulfide switch by using a 1:1, a 5:1, or a 10:1 ratio of opMPD to PDIA6. The percentage of clMPD was taken as measure of activity. (**B**) SDS-PAGE of four different samples was used to confirm the integrity of PDIA6 after incubation. (**C**) Purity of recombinant PDIs was verified by SDS PAGE. (**D**) RP-HPLC chromatogram of the conversion of opMPD to clMPD by reduced PDIs. Green line: non-treated opMPD; Black line: MPD treated with PDIA1; Dotted black line: MPD treated with PDIA3; Red line: MPD treated with PDIA6. (**E**) Histogram of the average PDI activities (relative to PDIA6) as calculated by peak areas from the data given in (**D**).

Surprisingly, PDIA6 displays only low isomerization activity for the opMPD-to-clMPD switch (Fig. 1A). Even a molar ratio of 1:10 [PDIA6]:[opMPD] produces only a slow rise (Fig. 1A). Consequently, lower concentration ratios were not tested. Since PDIA6 tends to degrade during storage at or above 4 °C, the integrity of the protein was routinely verified by SDS-PAGE upon incubation at 37 °C (Fig. 1B).

The low activity of PDIA6 when performing the thiol switch within the isolated opMPD suggests either that PDIA6 has no high affinity binding site to the isolated opMPD and/or that PDIA6 is not the principal specific isomerase that catalyzes the inactivation of ADAM17. For closer scrutiny, two additional PDIs (PDIA1 and PDIA3), which are also well-known as being located at the cell surface, were purified (Fig. 1C) and analyzed at several molar [PDI]:[opMPD] ratios (Fig. 1D and E). Figure 1D represents a characteristic reverse phase (RP)-HPLC chromatogram of such an analysis. The clMPD always elutes earlier than the opMPD (green line). Here, the same amounts of PDIs were tested with a molar ratio of [PDI]:[opMPD] = 1:10. PDIA1 (black line) converts more opMPD (right peak) to clMPD (left peak) compared with PDIA3 and PDIA6. The evaluation of these experiments revealed that PDIA1 catalyzes the disulfide switch 3.7-fold more efficiently then PDIA6, whereas the activity of PDIA3 lies in between those of PDIA1 and PDIA6 and is 1.4-fold higher than that of PDIA6.

### The differences in the activity between the tested PDIs might be a general characteristic

**Figure 2:**
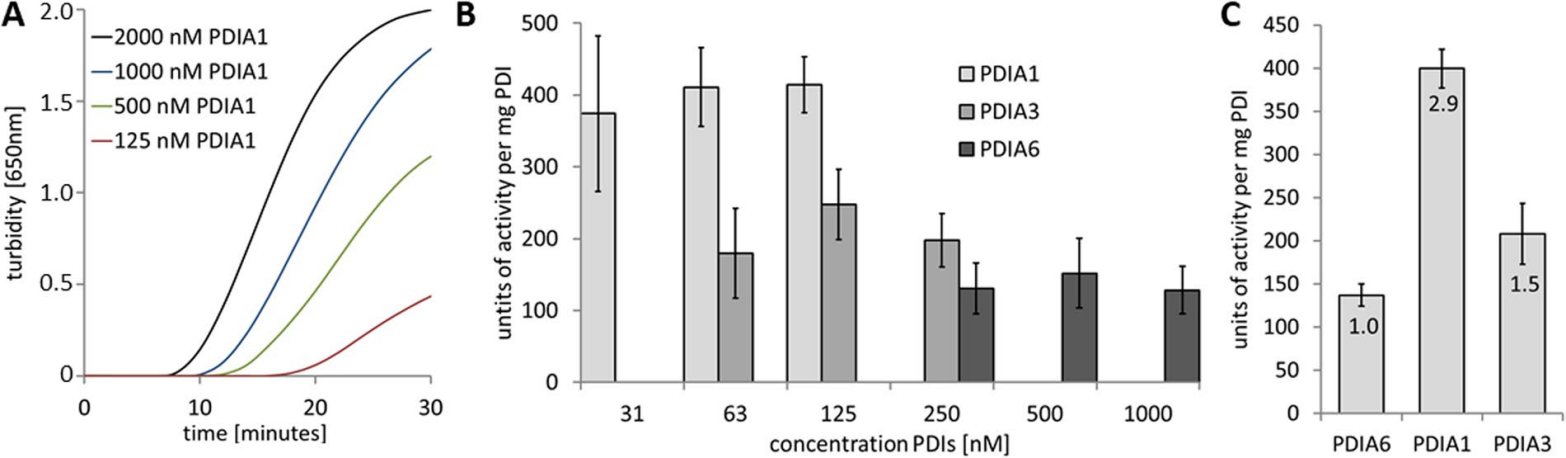
Insulin turbidity assay confirms general differences in the activity of PDIs with regard to disulfide isomerization. (**A**) Time-dependent increase in turbidity as a result of insulin precipitation attributable to the reduction of its disulfide bond catalyzed by PDIA1. The legend gives final PDI concentrations in the assay. (**B**) All three PDIs are able to catalyze the reduction of disulfide bonds in insulin, but higher amounts PDIA3 and PDIA6 than PDIA1 must be applied to obtain maximal precipitation. The indicated concentrations of PDIs were made up in a solution of 1 mg/ml insulin, and the enzyme activity was calculated as described in the methods section. (**C**) Display of the quantification and comparison of the capacities of PDIs to precipitate insulin.

An insulin turbidity assay was performed to establish whether the differences in activity are specific for the disulfide switch within the MPD or whether the purified PDIA1 comprises, in general, a higher isomerase activity than PDIA3 and PDIA6. In this assay, PDIs catalyze the reduction of disulfide bridges in insulin leading to its precipitation. This was detectable by an increase in turbidity recorded at λ = 650 nm, as presented for four different concentrations of PDIA1 in Fig. 2A. Higher amounts of PDIA3 and PDIA6 than of PDIA1 had to be applied in order to obtain a maximum of precipitation (Fig. 2B). The displayed concentrations were added to an insulin solution at a concentration of 1 mg/ml. The units of activity were calculated relative to 1 mg PDI, with 1 unit representing a change of 0.01 per minute in turbidity. Again, in this assay, PDIA1 was more active than PDIA3 and PDIA6, by 1.5-fold and 2.9-fold, respectively (Fig. 2C). These differences in activities are in agreement with previous reports^39,40^ and comparable with those with the opMPD as substrate. In order to catalyze the disulfide switch the PDIs have to be in their reduced state. Noteworthy, the differences in their activities were not due to incomplete reduction, which was exemplary verified by Ellman’s test (Table 1).

**Table 1:** Ellman’s tests confirmed the successful reduction of analysed PDIs. Upon DTT treatment and buffer exchanges, the percentage of reduced cysteine residues was tested by Ellman’s test. The concentrations of detected free disulfide groups were taken into the relation to the calculated concentration, which was taken from the protein concentration (measured by the absorption at 280 nm) multiplied by the numbers of cysteine residues in the individual PDIs. These concentrations were set to 100% of reduced cysteine residues. PDIA6 was tested in two experimental serials, ones together with PDIA1 and PDIA3, and ones with the indicated TRAP mutants. All tests were performed at least three times independently.

	mean value		mean value		mean value
PDIA1	90.4 ± 11.8	PDIA6	96.5 ± 25.3	PDIA6_C55SC58S	90.5 ± 21.7
PDIA3	77.4 ± 30.4	PDIA6_C58S	115.8 ± 30.4	PDIA6_2 TRAP	102.4 ± 32.2
PDIA6	100.3 ± 4.0	PDIA6_C193S	115.2 ± 15.5	PDIA6_inverse TRAP	126.1 ± 40.3

### The affinities of PDIs for their substrates are in a comparable nanomolar range

**Figure 3:**
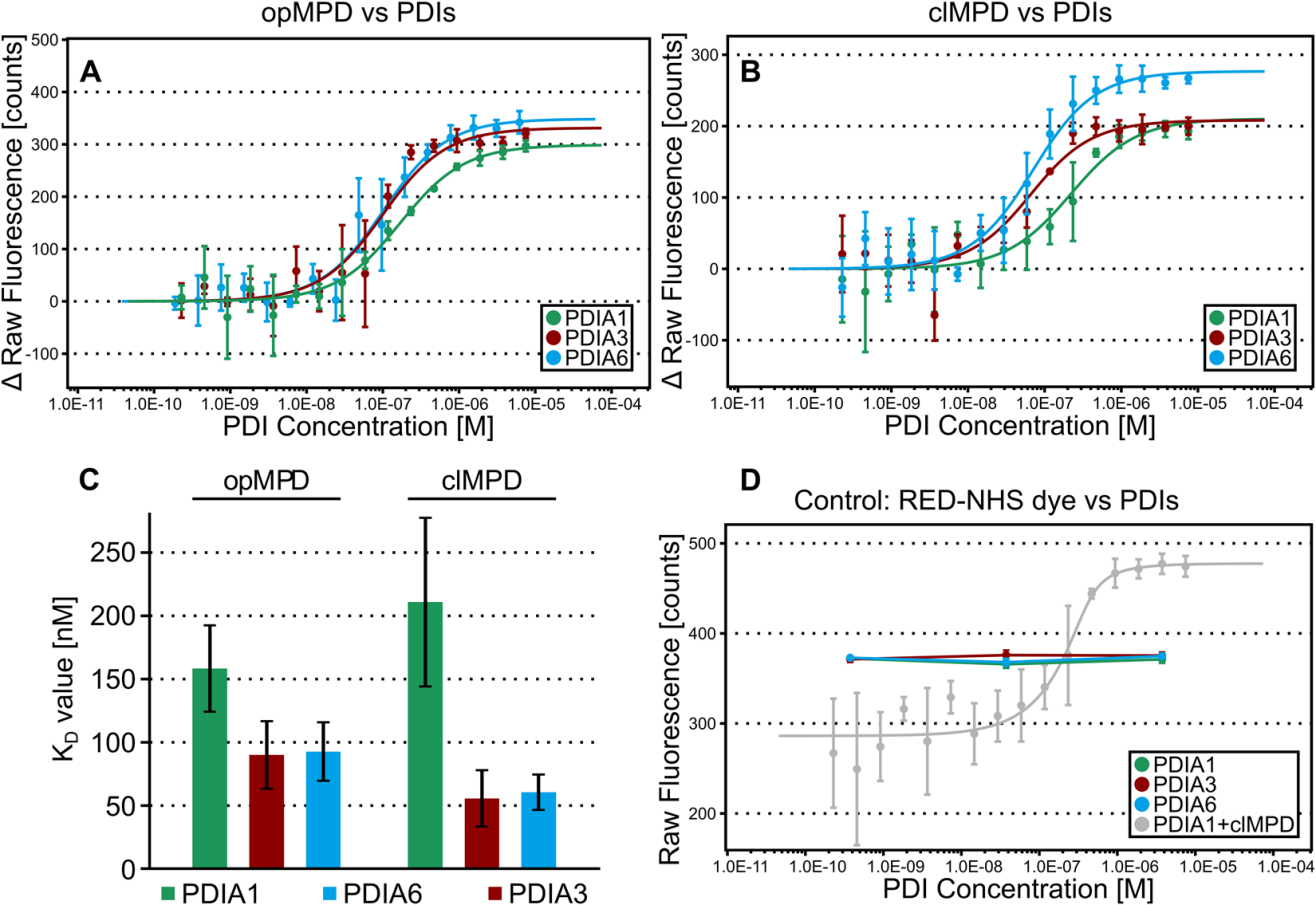
MST analysis of PDIs and MPD. (A and B) Dose-response curves of MST analysis (initial fluorescence, n = 3) of PDIA1, PDIA3 and PDIA6 with opMPD (**A**) or clMPD (**B**) as indicated in the caption. (**C**) Summary of the K_D_ values shown as column chart (error bars indicate respective 68% confidence intervals). The evident differences of the K_D_ values are not significant (95% confidence intervals overlap, not shown for clarity). (**D**) The dose-response curves of the initial fluorescence intensity of the control experiment to test for unspecific binding of PDIA1, PDIA3 and PDIA6 to the RED-NHS dye (3 concentrations, each n = 3, curve of PDIA1 against clMPD for reference in grey) show no PDI-concentration-dependent shift of the initial fluorescence.

High concentrations of PDIs are necessary to obtain measurable isomerization of the opMPD to its closed isomer. This implicates that PDIs interact with their substrates only with moderate affinities. To proof this implication the interactions between PDIs towards the MPD were measured by microscale thermophoresis (MST) experiments. The binding of PDIA1, PDIA3, and PDIA6 (see respective subsection below for details) to opMPD and clMPD was analyzed (Table 2 and Fig. 3). The dissociation constants K_D_ were, in all cases, in the same order of magnitude ranging from 50 to 200 nM and thus lower as previously reported K_D_ values of 1–10 µM in isomerization processes during protein folding^41^ or, in the case of PDIA6 and β3-integrin 21 µM, as an example for an extracellular disulfide switch^36^. No significant difference was observed for the affinity of PDIs towards the opMPD and the clMPD, indicating that the structural change attributable to the disulfide switch did not modify or block the interaction side for PDIs within the opMPD. These moderate affinities might be required for transient interactions with a fast release upon catalysis. Noteworthy, they are in the same order of magnitude as the affinity between MPD and GRP78, which protects the opMPD from the isomerization catalyzed by the PDIs^42^. Extracellular PDIA1, PDIA3, and PDIA6 share overlapping and complementary functions as shown for the activation of integrins during coagulation^34,36–38^. All three PDIs tested showed comparable affinities for the MPD of ADAM17, independently of its isoform. Hence, the local situation in the cells and/or tissues might determine which PDI is secreted in order to catalyze disulfide switches within extracellular targets. The complex task of identifying individual PDIs, which are secreted under particular situations, lies outside the scope of this report and will be addressed in future work.

**Table 2:** Dissociation constants (K_D_) of the PDI-MPD interaction with 68% confidence interval (CI) obtained by fitting the data from at least 3 independent MST analyses each with sixteen dilutions (1:2 dilution series) of the respective PDI at 25 °C. K_D_’s were calculated from the concentration-dependent ligand induced fluorescence intensity changes according to Eq. 1. Additional parameters resulting from the fit: Response amplitude of the fluorescence intensity shift between bound and unbound state, standard error (SE) of regression, reduced χ² and signal to noise.

Analyte	Ligand	K_D_ ± 68% CI [nM]	Response Amplitude [counts]	n	SE of Regression	Reduced χ²	Signal to Noise
PDIA1	opMPD	158 ± 34	298.9	3	19.75	0.45	16.25
	clMPD	211 ± 67	210.4	5*	20.17	2.05	11.21
PDIA3	opMPD	90 ± 27	331.5	3	30.26	2.24	11.77
	clMPD	55 ± 22	208.2	3	25.08	1.30	8.92
PDIA6	opMPD	93 ± 23	348.6	3	26.50	0.86	14.13
	clMPD	61 ± 14	276.9	3	19.41	1.30	15.32
PDIA6 C58S	opMPD	141 ± 38	411.6	3	34.58	3.73	12.79
	clMPD	113 ± 31	198.2	3	16.87	0.69	12.62
PDIA6 C193S	opMPD	142 ± 30	400.5	3	26.11	4.74	16.48
	clMPD	91 ± 55	155.4	3	28.86	2.47	5.78
PDIA6 C58S C193S	opMPD	192 ± 40	406.4	3	25.50	3.87	17.12
	clMPD	151 ± 40	148.2	3	11.96	0.26	13.31
PDIA6 C55S C58S	opMPD	138 ± 27	447.3	3	26.57	22.13	18.09
	clMPD	191 ± 88	171.5	3	24.15	1.15	7.63
PDIA6 C55S C190S	opMPD	115 ± 20	314.3	3	16.59	8.73	20.35
	clMPD	116 ± 42	178.2	3	19.79	0.74	9.67

*Replicates increased to 5 due to variations at low concentration.

### The order of the PDI domains

**Figure 4:**
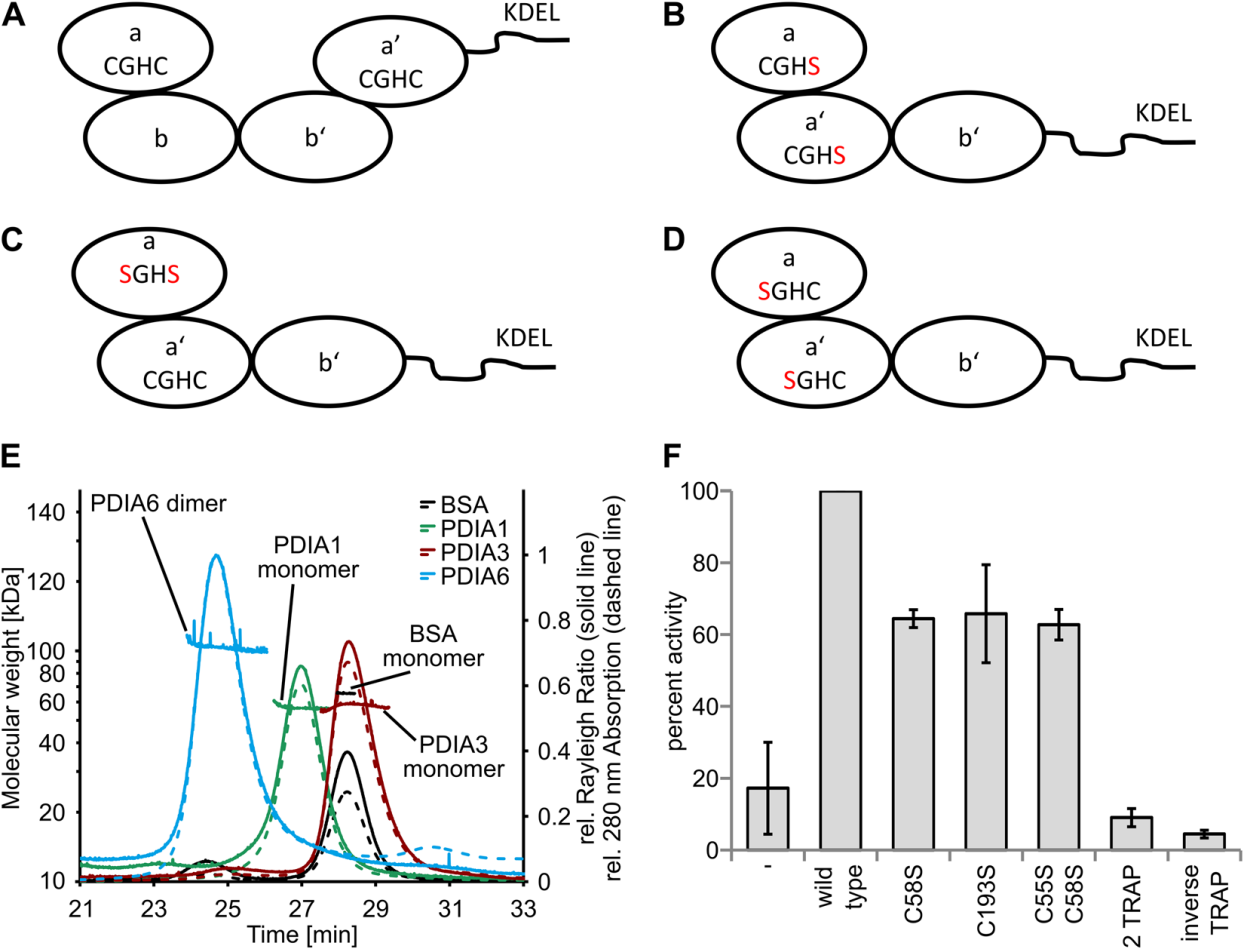
(**A**) Schematic drawing of the domain arrangement of PDIA1 and PDIA3 in which the CGHC-motive of the catalytically active sites are indicated. (**B**) Schematic domain arrangement of the 2 TRAP mutant of PDIA6, where both C-terminal cysteine residues of the CGHC-motive are exchanged to serine residues. (**C**) Schematic drawing of the C55SC58S mutant and (**D**) the inverse TRAP mutant bearing exchanges in the N-terminal cysteine residues of the CGHC-motive. (**E**) Chromatograms of the SEC-MALS analysis of PDIA1, PDIA3, PDIA6 and BSA as control. Shown is only the relevant section of the complete elution profile (60 minutes at 0.5 ml/minutes) of the detected absorption at 280 nm (dashed line) and the Rayleigh ratio (solid line, derived from MALS) normalized to PDIA6. In addition, the calculated molecular weight for each slice of the analyzed peaks is shown. (**F**) Comparison of the activities of indicated TRAP mutants relative to wild-type PDIA6 whose activity was set to 100%.

We further focused on the active sites of the PDIs, which contain a typical CGHC-motive. The N-terminal cysteine residue is thought to be responsible for the reduction and isomerization of disulfide bonds through intermediate intermolecular disulfide bond formation. The second cysteine residue is thought to resolve mixed disulfides of PDIs and substrates^43^. PDIA1 and PDIA3 have four domains arranged in the order: the first catalytic domain a, two catalytic inactive b and b’ domains, followed by the second catalytically active a’ domain (Fig. 4A). In contrast, PDIA6 consists of only three domains, arranged in the order a, a′, and b domain, and lacks the b′ domain^28^ (see Fig. 4B–D). PDIA1 (56.71 ± 0.57 kDa, theoretical M_w_ monomer: 57.6 kDa) and PDIA3 (57.7 ± 4.6 kDa, theoretical M_w_ monomer: 56.6 kDa) appear to be monomeric in solution as determined by size-exclusion chromatography (SEC)-Multi angle laser light scattering (MALS) analysis (Fig. 4E). The *in vivo* dimerization of PDIA1 supposedly functions as a regulatory mechanism in the ER and occurs through a bb’ dimer^44^. *In vitro*, dimerization is dependent on the presence of divalent cations^45^ and leads to a decreased activity^46^. In contrast, PDIA6 exist as a dimer in solution (103.7 ± 8.3 kDa, theoretical M_w_ monomer: 48.8 kDa) and is most likely active as such. This suggests, that the PDIA6 lacking the b’ and being present as a dimer, is likely differently regulated than PDIA1 and presumably PDIA3.

### The catalytic domains of PDIA6 contribute equally to the disulfide switch and require full integrity

**Figure 5:**
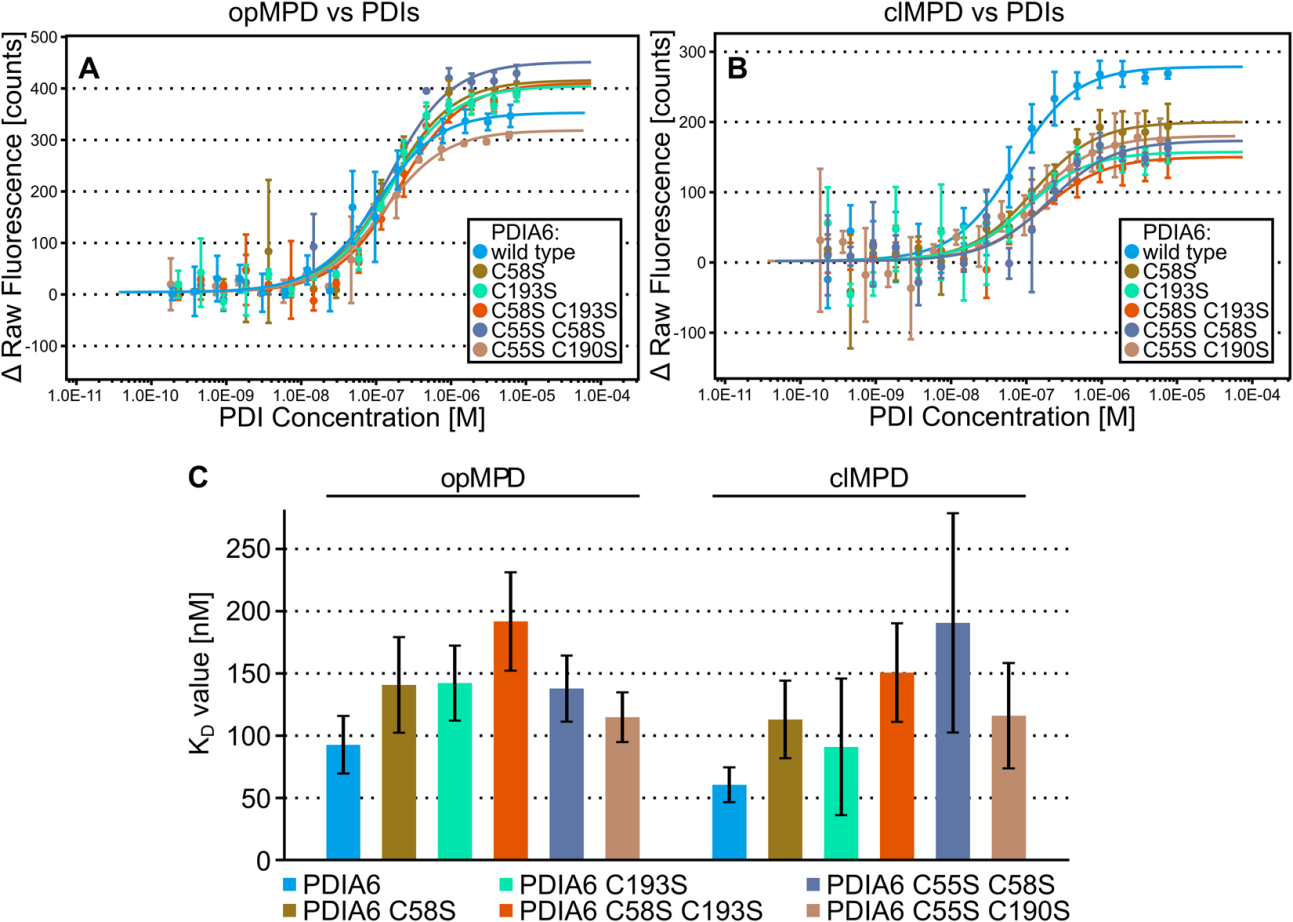
MST analysis (initial fluorescence, n = 3) of PDIA6 TRAP mutants with opMPD (**A**) or clMPD (**B**). Respective dose-response curves are indicated in the caption. (**C**) Corresponding K_D_ values are summarized in a column chart (the error bars indicate respective 68% confidence intervals).

So-called TRAP mutants were generated to analyze the properties of the catalytic sites within PDIA6. In these mutants, the cysteine residues are exchanged by serine residues (Fig. 4B). Consequently, a disulfide bridge covalently traps mixed disulfide intermediates of PDI and substrate, which are normally unlinked by the second resolving cysteine residue. Isomerization processes in which the intermediates are unlinked by the substrates should be unaffected. The latter situation appears to be likely for the disulfide switch in ADAM17. In this case, the classic TRAP mutants should comprise a comparable isomerization activity to that of the wild-type enzyme. The replacement of the second cysteine residue in the CGHC-motive in the a or a’ domains results in the classic C58S or C193S TRAP mutants. Additional to the single TRAP mutants, the double TRAP mutant (2 TRAP) with an exchange in C58S and C193S was generated (Fig. 4B). Both single mutants showed comparable reduced activities in catalyzing the disulfide switch of approximately 60% of the wild-type PDIA6 (Fig. 4F). In addition, the 2 TRAP mutant, with the C58S and C193S exchange, was completely inactive. Moreover, the a and a’ domain catalyzes the isomerization with the same efficiency, and so no major difference might occur in their interaction with the opMPD. A residual activity of the N-terminal attacking cysteine residue can be excluded because the activity of the single TRAP mutants was on the same level as the activity of a PDIA6 variant in which both cysteine residues of the a domain were exchanged (C55S C58S, Fig. 4C). Because of the inactivity of the classic TRAP mutants, an inverse TRAP mutant was generated to ensure that the C-terminal resolving cysteine residue did not catalyze the disulfide switch (Fig. 4D). This mutant contains an exchange of C55S and C190S and is completely inactive, like the 2 TRAP mutant. Remarkably, all N- and C-terminal exchanges of the cysteine residues in the CGHC-motive result in inactive enzymes with regard to the disulfide switch within the opMPD. Thus, a definite identification of the attacking residue is not possible; a change in the redox potential of the active site caused by the mutations appears as the most probable cause for the inactivity of the TRAP mutants. Again, to exclude the possibility that the reduced activity of the TRAP mutants is due to an incomplete reduction, Ellman’s tests were performed exemplary for the wild type PDIA6 and all mutants, whereby no significant differences in the percentage of reduction was detected (Table 1). Noteworthy, the cysteine to serine exchanges did not alter the ability of the isomerase to bind the MPD, since all variants and the wild type isomerase comprise comparable K_D_-values (Fig. 5). Hence, the cysteine residues of the active sites have no notable impact on the affinity between PDIA6 and the MPD.

In conclusion, all PDIs tested here have basic affinities for potential substrate proteins in a similar nanomolar range. However, in order to produce a general statement, more interactions and affinities have to be tested. Moderate binding affinities to certain proteins allow rapid catalysis and the release of these proteins, which then follow their fate. Regarding ADAM17, the moderate affinity might permit guiding interactions such as its transport to the cell surface by iRhoms^47–51^. The integrity of catalytic domains is essential for activity, possibly to ensure the correct redox potential. The finding of no notable difference in the activities of the catalytic domains, at least for PDIA6, further supports the hypothesis of a moderate general interaction. Thus, we consider it reasonable that there is no unique principal isomerase of ADAM17. Instead, the type of PDI released to the cell surface probably decides which isomerase catalyzes the disulfide switch, and the underling regulatory mechanism of this transport acts to fine-tune the activity of ADAM17.

## Methods

### Recombinant Proteins

DNAs coding for mature human protein disulfide isomerases (PDIs), PDIA1 (MW 57.6 kDa), PDIA3 (MW 56.6 kDa), and PDIA6 (MW 48.8 kDa) were cloned into pET28. TRAP mutants of PDIA6 (C58S, C193S, 2 TRAP: C58SC193S, C55SC58S, inverse TRAP C55SC190S) were generated by overlapping extension PCR cloning. Protein expression was performed in *E. coli* BL21 (DE3) by inducing the culture at about OD_600_ = 0.8 with 1 mM isopropyl-β-D-1-thiogalactopyranoside at 37 °C for three hours. Bacteria were pelleted and resuspended in phosphate buffer saline (PBS). A protease inhibitor cocktail (cOmplete; Roche Diagnostics) and Benzonase (Santa Cruz Biotechnologies) were added before cell lysis and freezing at −20 °C. Bacteria were lysed by sonification, and PDIs were purified via Ni-affinity and size-exclusion chromatography (SEC) by using HisTrap and Superdex 75 or 200 16/60 columns (GE Healthcare). Recombinant open membrane-proximal domain (opMPD) (9.9 kDa) was obtained in a comparable procedure but was further purified via reverse-phase (RP) HPLC as described earlier^11^. The ectodomain of ADAM17 was obtained from Sigma-Aldrich. Human interleukin-6 (IL-6) was expressed and purified as described previously^52^.

### Reduction of PDIs

PDIs were incubated with 50 mM dithiothreitol (DTT) for 120 minutes at room temperature in order to reduce the proteins prior to isomerization experiments. Subsequently, DTT was removed by using NAP^TM^−5 and NAP^TM^-10 gel filtration columns (GE Healthcare) according the manufacturers’ instructions by using PBS. Gel filtration was performed twice, and the removal of DTT was exemplarily tested by Ellman’s test. The protein concentration was determined by absorbance at 280 nm.

### Ellmann’s test

To verify that the disulfide bridges were reduced upon DTT treatment and buffer exchange Ellman’s tests were performed. 200 µl Ellman’s reagent (50.0 mM sodium acetate, 2.0 mM 5,5′-Dithio-bis (2-nitrobenzoic acid)) were mixed with 500 µl ddH_2_O, 100 µl 1 M Tris pH 8.0 and 200 µl of either standard or sample probes. Standard curve includes a blank sample (PBS) and samples containing β-mercaptoethanol from 1 to 16 µM in PBS. After preparation of samples, they were incubated for 5 minutes at room temperature before measuring the absorption at 412 nm. To calculate the concentration of free thiol groups an extinction of 13 600 M^−1^cm^−1^ was taken. The theoretical concentrations of free disulfide groups were calculated from the molar concentrations calculated from the absorptions of the protein solutions at 280 nm, which was multiplied by the theoretical number of cysteine residues (PDIA1: 6; PDIA3 7; PDIA6: 6; C58S and C193S: 5, 2 TRAP, C55SC58S and inverse TRAP: 4). The calculated number of free disulfide groups was set to 100% reduced disulfide groups and from the actual measured concentration the percentage of reduced disulfide groups were calculated. All PDIs and variants of PDIs were tested at least three times.

### Isomerization Experiments

Ten micrograms of opMPD were incubated together with freshly reduced PDIs in PBS with molar ratios as indicated in the results section. If not otherwise indicated, the isomerization was stopped after 170 minutes at 37 °C by addition of 0.1% trifluoroacetic acid (TFA) and analyzed by RP HPLC (Bio-200-C18 5 µ, MultoHigh, CS-Chromatography Service GmbH). Time kinetics were measured for PDIA6 at a ratio of 1:1, 1:5, and 1:10 PDIA6 to opMPD. For comparison of the activities of PDIs, PDIA1 was used in ratios 1:10, 1:20, and 1:30, PDIA3 in ratios 1:5, 1:10, and 1:20, and PDIA6 in ratios 1:5 and 1:10. Peak areas of the opMPD and the closed MPD (clMPD) were determined, and the sum was set to 100% total MPD. The percentage of the closed isoform was taken as a measure of PDI activity. The means and the standard deviations of at least three independent experiments were calculated. Since PDIA6 tends to degrade, the integrity of PDIs were routinely tested by a SDS-PAGE after incubation at 37 °C for the longest time of incubation.

### Insulin turbidity assay

The activity of PDIs to catalyze the reduction of disulfide bridges within insulin was analyzed in a solution of 1 mg/ml insulin in 100 mM Tris-HCl pH 7.5, 5 mM MgCl_2_, and 0.5 mM ATP, which was incubated with the indicated amounts of PDI in the presence of 1 mM DTT at 25 °C. Turbidity was detected at 650 nm against reference samples without PDIs. One unit represents the change of 0.01 OD_650_ per minute per 1 mg protein.

### SEC-MALS analysis

The SEC-multi angle light scattering (MALS) measurements were performed using an online MALS detector (miniDAWN Treos, Wyatt Technology Corp.) coupled to an Agilent Technologies 1100 series HPLC system equipped with a refractive index detector (differential refractometer, RID, G1362A, Agilent Technologies) and a multiple wavelength detector (DAD, G1315B, Agilent Technologies). Protein samples (100–500 µg/analysis) were separated on a Superdex 200 10/300 GL SEC column (GE Healthcare Life Sciences) at a flow rate of 0.5 ml/minute in PBS pH 7.4. The MALS detector was normalized with bovine serum albumin (BSA) (Sigma-Aldrich) prior PDI analysis. The molecular weight was calculated based on the MALS and refractive index data by using 0.185 as refractive index increment (dn/dc) and a second virial coefficient of zero by using the Zimm plot. Data acquisition and analysis was performed with the software ASTRA version 5.3.4.10 (Wyatt Technology Corp.).

### Microscale thermophoresis (MST) measurements

MST was performed to measure the binding affinity (*K*_D_ value) of clMPD or opMPD and PDIA1, PDIA3, PDIA6 (incl. variants). The fluorescence labeling of the MPDs was performed with the amine-reactive MO-L003 Monolith™ Protein Labeling Kit RED-NHS (NanoTemper Technologies) according to the manufacturer’s recommended standard protocol. The unlabeled binding partner (PDIs) was titrated (7.5 μM to 0.9537 nm, 1: 2 dilution series) against a constant MPD concentration (20 nM) in PBS-P buffer (PBS, 0.01% P20 at pH 7.4). The measurement was performed in triplicates with a Monolith NT.115 instrument by using MO-K002 Monolith™ NT.115 Standard Treated Capillaries (NanoTemper Technologies) with 40% (clMPD) or 50% (opMPD) LED power and ‘medium’ MST power at 25 °C by using MO.Control Software v1.4.3. The interaction of the MPDs with the PDIs resulted in a dose-dependent ligand-induced fluorescence intensity shift as verified by the recommended SDS-denaturation test (User Guide NT.115; NanoTemper Technologies). Briefly, the first and last three samples of the total 16 samples per measurement were centrifuged (10 minutes, 15 000 ***g***) to remove potentially precipitated protein causing the observed shift. Next, the supernatant was incubated for 5 minutes at 95 °C including 2% (w/v) sodium dodecyl sulfate and 20 mM DTT. The fluorescence intensity of these samples was measured with the Monolith NT.115 instrument as described above, resulting in the same fluorescence intensity (≤±10%) in all tested samples. Unspecific binding to the RED-NHS dye was tested in triplicates by measuring PDIA1, PDIA3 and PDIA6 (each at 3 dilutions of 3750, 37.5 and 0.375 nM) against 20 nM of the dye alone with the same instrument settings as those described above in PBS-P. The reactive NHS-ester of the dye was inactivated by incubation with a fourfold molar excess of ethanolamine for 30 minutes at room temperature. The MST analysis and the calculation of the *K*_D_ values based on the change of the initial fluorescence intensity were performed by using MO. affinity analysis software v2.2.4 (NanoTemper Technologies) to fit the following equation:

1 $$f({C}_{PDI})={R}_{U}+\frac{({R}_{B}-{R}_{U})({C}_{PDI}+{C}_{MPD}+{K}_{d}-\sqrt{{({C}_{PDI}+{C}_{MPD}+{K}_{d})}^{2}-4{C}_{PDI}{C}_{MPD}}}{2{C}_{MPD}}$$

with *C*_PDI_ = concentration of PDI, *C*_MPD_ = concentration of MPD, *R*_U_ = response value of unbound state, *R*_B_ = response value of bound state, and *K*_D_ = dissociation constant.
